# CRISPR-FMC: a dual-branch hybrid network for predicting CRISPR-Cas9 on-target activity

**DOI:** 10.3389/fgeed.2025.1643888

**Published:** 2025-08-29

**Authors:** Chuxuan Li, Jian Li, Quan Zou, Hailin Feng

**Affiliations:** ^1^ School of Mathematics and Computer Science, Zhejiang A&F University, Hangzhou, China; ^2^ Yangtze Delta Region Institute (Quzhou), University of Electronic Science and Technology of China, Quzhou, China; ^3^ Institute of Fundamental and Frontier Sciences, University of Electronic Science and Technology of China, Chengdu, China

**Keywords:** CRISPR-Cas9, RNA-FM, deep learning, on-target activity, SgRNA

## Abstract

**Introduction:**

Accurately predicting the on-target activity of sgRNAs remains a challenge in CRISPR-Cas9 applications, due to the limited generalization of existing models across datasets, small-sample settings, and complex sequence contexts. Current methods often rely on shallow architectures or unimodal encodings, limiting their ability to capture the intricate dependencies underlying Cas9-mediated cleavage.

**Methods:**

We present CRISPR-FMC, a dual-branch hybrid neural network that integrates One-hot encoding with contextual embeddings from a pre-trained RNA-FM model. Multi-scale convolution (MSC), BiGRU, and Transformer blocks are employed to extract hierarchical sequence features, while a bidirectional cross-attention mechanism with a residual feedforward network enhances multimodal fusion and generalization.

**Results:**

Across nine public CRISPR-Cas9 datasets, CRISPR-FMC consistently outperforms existing baselines in both Spearman and Pearson correlation metrics, showing particularly strong performance under low-resource and cross-dataset conditions. Ablation experiments confirm the contribution of each module, and base substitution analysis reveals a pronounced sensitivity to the PAM-proximal region.

**Discussion:**

The PAM-proximal sensitivity aligns with established biological evidence, indicating the model’s capacity to capture biologically relevant sequence determinants. These results demonstrate that CRISPR-FMC offers a robust and interpretable framework for sgRNA activity prediction across heterogeneous genomic contexts.

## 1 Introduction

The CRISPR-Cas9 (Clustered Regularly Interspaced Short Palindromic Repeats and CRISPR-associated protein 9) system is an adaptive immune mechanism originating from bacteria and archaea (Roberts and Barrangou, 2020). In recent years, it has emerged as a powerful tool for precise genome editing (Doudna and Charpentier, 2014). This system primarily relies on single-guide RNA (sgRNA) (Meier et al., 2017) to direct the Cas9 endonuclease to specific genomic loci, where it introduces double-strand breaks (DSBs) (Jinek et al., 2012). These breaks activate endogenous DNA repair pathways, resulting in insertions or deletions (indels) at the target site (Cong et al., 2013). Owing to its high editing efficiency, programmable specificity, and operational simplicity, CRISPR-Cas9 has been widely applied in gene function analysis, hereditary disease research, and genomic engineering (Shalem et al., 2014).

However, the activity of sgRNAs varies substantially across different target sequences and cell types, leading to inconsistencies in editing efficiency and experimental reproducibility (Doench et al., 2016). This variability underscores the necessity of developing accurate computational models to predict sgRNA on-target activity, thereby facilitating the rational design of highly effective sgRNAs and improving the reliability of genome editing experiments (Haeussler et al., 2016).

Computational methods for predicting on-target sgRNA activity are typically classified into two major categories: conventional approaches based on heuristic rules or classical machine learning algorithms, and more recent methodologies employing deep learning frameworks.

Traditional predictive models typically depend on manually engineered features—such as nucleotide frequency, GC content, and inferred secondary structures (Bochman et al., 2012)—and utilize algorithms like support vector machines (SVMs) (Vishwanathan and Murty, 2002) and logistic regression Hosmer (Hosmer Jr et al., 2013). Notable representatives of this category include the Rule Set family, WU-CRISPR (Wong et al., 2015), and CRISPRscan (Moreno-Mateos et al., 2015). Although these approaches provide a certain level of interpretability, their reliance on predefined features restricts their capacity to model intricate sequence characteristics and long-range contextual information.

In contrast to traditional approaches, deep learning methods, empowered by end-to-end representation learning capabilities, can automatically extract high-order features from large-scale screening data and have demonstrated significant advantages in sgRNA on-target efficiency prediction tasks (Chuai et al., 2018). Existing approaches can be broadly classified into four main categories based on their model architectures and learning strategies. Firstly, models based on convolutional neural networks (CNNs) (Alzubaidi et al., 2021) are widely applied for local sequence pattern modeling. For instance, DeepCas9 (Kim et al., 2019) employs fixed-length convolutional kernels to extract localized nucleotide fragment features. Building upon this idea, CRISPR_HNN (Li et al., 2025) integrates a multi-scale convolutional module (MSC) (Jin et al., 2022), thereby enhancing the model’s capacity to capture local sequence patterns across diverse receptive fields. Secondly, to improve the identification of key functional bases, some methods integrate attention mechanisms. CRISPR-ONT (Zhang et al., 2021) and AttnToCrispr-CNN (Liu et al., 2019) apply attention modules to emphasize important base positions, thereby improving overall modeling and predictive performance. Thirdly, recurrent neural networks (RNNs) (Dhruv and Naskar, 2020) have been adopted to capture sequential and contextual dependencies. For instance, CrnnCrispr (Zhu et al., 2024) leverages Bidirectional Gated Recurrent Unit (BiGRU) modules (Yan et al., 2021) to enhance the modeling of contextual relationships along the sgRNA sequence. Lastly, to further strengthen structural modeling and cross-dataset generalization, several studies have explored more expressive architectures. TransCrispr (Wan and Jiang, 2022) employs a Transformer (Han et al., 2021) module to improve long-range dependency modeling, while C-RNNCrispr (Zhang et al., 2020) integrates external biological features to enhance performance across diverse data distributions.

Although recent deep learning methods have achieved promising results on various public CRISPR-Cas9 datasets, several key limitations remain. (1) Most existing encoding strategies are overly simplistic, failing to jointly capture shallow compositional and deep contextual features within sgRNA sequences. (2) Many feature extraction frameworks lack the architectural capacity to concurrently model both local sequence motifs and long-range dependencies. (3) Multimodal fusion strategies are typically shallow and semantically under-aligned, limiting model expressiveness and generalization, especially under complex sequence distributions or small-sample scenarios.

In response to these challenges, we propose CRISPR-FMC, an innovative dual-branch deep neural network with a novel structural design, systematically optimized along three key architectural dimensions: Pre-training, Multimodal Processing, and Cross-modal Interaction. (1) In the Pre-training stage, CRISPR-FMC integrates one-hot encoding and RNA-FM pre-trained embeddings (Chen et al., 2022) to construct two complementary input branches. The one-hot pathway captures low-level nucleotide composition, while the RNA-FM pathway encodes high-level contextual semantics, enabling the model to learn multi-level sequence representations from the outset. (2) During Multimodal Processing, each branch undergoes independent transformation through a sequence of modules specifically designed for its input characteristics. In particular, a multi-scale convolution (MSC) block extracts local motifs of varying lengths, and the Transformer and BiGRU layers are responsible for modeling long-range dependencies and sequential relationships. This parallel architecture enhances the model’s capacity to capture hierarchical and complementary features within each modality. (3) In the Cross-modal Interaction module, a bidirectional cross-attention mechanism (Chen et al., 2021) facilitates semantic alignment and feature-level integration between the two branches. The resulting fused features are subsequently refined by a residual feedforward network (FFN) (Xie S. et al., 2023), enabling high-order nonlinear transformations and improving the model’s generalization capability. Extensive evaluations across nine publicly available CRISPR-Cas9 benchmark datasets show that CRISPR-FMC consistently outperforms state-of-the-art baseline models in both predictive accuracy and robustness. To enhance practical utility, we developed an interactive web-based visualization platform that provides real-time interpretability and supports efficient sgRNA design.

The core contributions of this work can be summarized as follows:

1. We propose a dual-branch multimodal encoding scheme that integrates One-hot encoding and RNA-FM pre-trained embeddings, enabling the model to jointly capture low-level base composition and high-level contextual semantics. This design facilitates multi-level representation of sgRNA sequences and enhances the expressiveness of input features.
2. We construct a hybrid feature extraction framework that incorporates MSC modules for local motif detection, together with independent Transformer and BiGRU components for modeling long-range dependencies. This integration enables the model to simultaneously capture fine-grained local patterns and broader global sequence dependencies.
3. We design a Cross-modal Interaction mechanism that integrates a bidirectional cross-attention module and a FFN, which promotes deep semantic alignment and nonlinear feature fusion between modalities, improving representation stability and generalization capacity.
4. We conducted a comprehensive evaluation of the proposed method across nine publicly available CRISPR-Cas9 benchmark datasets. The results demonstrate that CRISPR-FMC consistently outperforms state-of-the-art approaches in both predictive accuracy and cross-dataset generalization, exhibiting particularly strong performance under limited-sample conditions.

## 2 Materials and methods

### 2.1 Datasets

To comprehensively evaluate the predictive performance of the CRISPR-FMC model across diverse experimental settings and sample sizes, nine publicly available CRISPR-Cas9 on-target efficiency datasets were employed. These datasets span multiple Cas9 variants, various cell types, and high-throughput screening experiments of different scales. All datasets are widely adopted in prior studies and serve as benchmark resources for modeling CRISPR-Cas9 on-target activity.

The datasets are categorized into three groups based on sample size (see Table 1):

**TABLE 1:** A comprehensive summary of the CRISPR-Cas9 on-target efficiency datasets employed in this study. Detailed information, including Cas9 variants, cell lines, and experimental methods, are provided in Supplementary Table 4.

Dataset	Total samples	Scale level	Source
WT	55,603	Large-scale	Wang et al. (2019)
ESP	58,616	Large-scale	Wang et al. (2019)
HF	56,887	Large-scale	Wang et al. (2019)
xCas9	37,738	Medium-scale	Kim et al. (2020)
SpCas9-NG	30,585	Medium-scale	Kim et al. (2020)
Sniper-Cas9	37,794	Medium-scale	Kim et al. (2020)
HCT116	4,239	Small-scale	Hart et al. (2015)
HELA	8,101	Small-scale	Hart et al. (2015)
HL60	2,076	Small-scale	Wang et al. (2014b)

Large-scale datasets. The WT (Wang et al., 2019), ESP (Wang et al., 2019), and HF (Wang et al., 2019) datasets comprise 55,603, 58,616, and 56,887 sgRNA sequences, respectively. These datasets originate from genome-wide screening experiments conducted by Wang et al. (2019) using SpCas9 and its high-fidelity variants (e.g., eSpCas9(1.1), SpCas9-HF1). Due to their broad genomic coverage and substantial sequence diversity, they serve as ideal benchmarks for evaluating model performance and stability under large-sample conditions.

Medium-scale datasets. The xCas9 Kim et al. (2020), SpCas9-NG (Kim et al., 2020), and Sniper-Cas9 Kim et al. (2020) datasets contain 37,738, 30,585, and 37,794 sgRNA sequences, respectively. These datasets were derived from experiments summarized in Kim et al. (2020), where predictive models were built for a series of PAM-variant SpCas9 systems.

Small-scale datasets. The HCT116 (Hart et al., 2015), HELA (Hart et al., 2015), and HL60 (Wang T. et al., 2014) datasets contain 4,239, 8,101, and 2,076 sgRNA sequences, respectively. These datasets were originally compiled by Chuai et al. (2018), who integrated four independently validated CRISPR screening datasets involving 1,071 genes across multiple human cell lines. We selected three of these cell line-specific datasets—HCT116, HELA, and HL60—for our experiments. Redundant entries were removed to ensure non-overlapping, high-confidence training samples.

Each sample consists of a 23-nucleotide sequence, comprising a 20-nt sgRNA protospacer and a 3-nt PAM (e.g., NGG) located at the 3′ end. The sgRNA guides the Cas9 protein to the target site via complementary base pairing, while the PAM—though not directly involved in hybridization—is essential for Cas9 binding and cleavage. To preserve the integrity of both sequence context and cleavage specificity, the full 23-nt sgRNA-PAM sequence was used as input to our model, enabling richer representation learning.

To maintain consistency across datasets originating from heterogeneous experimental conditions, all indel frequencies were scaled to the [0,1] interval through Min-Max normalization (Saini et al., 2024). Table 1 summarizes the Cas9 variant, cell type, and sample size associated with each dataset. Model training and evaluation were conducted based on a five-fold cross-validation protocol. In each fold, 80% of the data served as the training set and the remaining 20% as the test set; additionally, 10% of the training data was set aside as a validation subset for hyperparameter tuning and early stopping. This evaluation strategy ensures robust and unbiased performance estimation across datasets with varying distributions.

### 2.2 Sequence encoding

**FIGURE 1:**
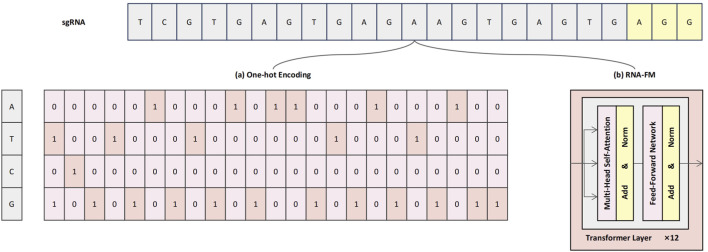
Schematic illustration of the dual-branch sequence encoding framework. **(a)** One-hot encoding represents each nucleotide as a sparse four-dimensional binary vector, preserving base identity and positional specificity. **(b)** RNA-FM embeddings provide high-level contextual representations, extracted from a pre-trained Transformer-based language model.

To comprehensively characterize sgRNA sequences and enhance the model’s capacity to capture both fine-grained local patterns and broader contextual dependencies, we propose a dual-branch sequence encoding strategy that integrates conventional one-hot encoding with semantic embeddings derived from a pre-trained RNA-FM module, as illustrated in Figure 1. This hybrid representation framework is designed to provide the model with both interpretable low-level compositional signals and enriched high-level contextual semantics, thereby enhancing predictive performance and feature expressiveness (Song X. et al., 2024).

Specifically, the One-hot encoding branch (Figure 1a) preserves the exact nucleotide composition of each base in the sgRNA sequence. In this scheme, each nucleotide—A, T, G, and C—is represented by a four-dimensional One-hot vector: A = [1, 0, 0, 0], T = [0, 1, 0, 0], C = [0, 0, 1, 0], and G = [0, 0, 0, 1]. As a result, the full 23-nucleotide sgRNA sequence—comprising a 20-base protospacer and a 3-base PAM—is encoded as a 23×4 binary matrix. This sparse yet structured format is particularly well-suited for convolutional modules, enabling the model to effectively capture local sequence motifs and positional dependencies.

However, One-hot encoding alone lacks the capacity to capture semantic relationships or contextual dependencies among nucleotides. To address this limitation, RNA-FM embeddings are incorporated as a complementary representation (Figure 1b). RNA-FM is a Transformer-based RNA language model that was pre-trained on a large-scale corpus of over 240 million non-coding RNA sequences, capable of modeling rich contextual semantics through its core self-attention mechanisms (Huang et al., 2023). This allows the model to learn complex relationships between nucleotides that are distant in the primary sequence, thereby implicitly capturing information regarding potential secondary structures and other conserved functional motifs. During encoding, the mean vector from the final Transformer module is extracted to produce a 640-dimensional dense embedding that encapsulates these learned long-range dependencies, structural preferences, and contextual sequence features.

By integrating the explicit base identity information from One-hot encoding with the context-aware semantic representations provided by RNA-FM, the proposed dual-branch encoding scheme enables complementary feature fusion at both compositional and semantic levels. This multimodal sequence representation (Xie J. et al., 2023) allows the subsequent network modules to jointly learn localized structural features and global contextual dependencies, thereby improving the model’s ability to accurately predict sgRNA on-target editing efficiency (Xiang et al., 2021).

### 2.3 Model

Recent advances in deep learning have catalyzed the development of a diverse array of models designed to improve the accuracy and robustness of CRISPR-Cas9 on-target activity prediction (Elkayam and Orenstein, 2022; Bao and Liu, 2024; Zhu et al., 2024). However, existing methods still face significant challenges in modeling the high-dimensional structure of biological sequences and effectively integrating multimodal information. Specifically, the sgRNA sequence encodes hierarchical semantic features (Sun et al., 2024) that are difficult to fully represent through a single encoding scheme. In addition, local pattern recognition and global dependency modeling are often treated independently, which hinders joint optimization. Furthermore, the absence of deep semantic alignment (Ates et al., 2023) during heterogeneous modality fusion can lead to feature redundancy and semantic inconsistency, ultimately limiting predictive performance.

**FIGURE 2:**
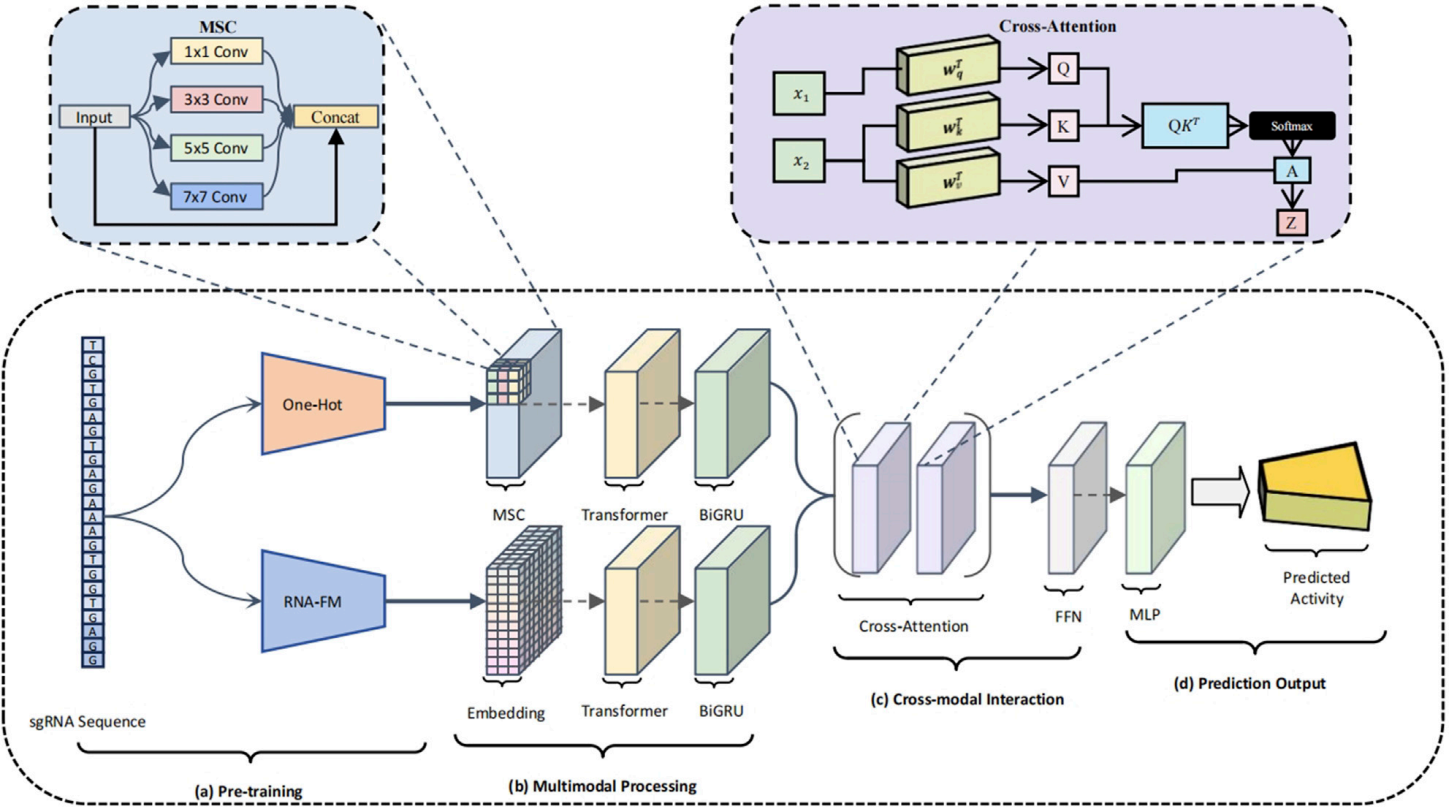
Schematic overview of the CRISPR-FMC model architecture. The model consists of four key modules: **(a)** Pre-training, where the sgRNA sequence is encoded through both one-hot vectors and RNA-FM embeddings, forming a dual-channel input that captures structural and semantic information; **(b)** Multimodal Processing, which applies MSC modules, Transformer encoders, and BiGRU units within each branch to extract local structural patterns and global contextual dependencies; **(c)** Cross-modal Interaction, which enables deep semantic fusion between modalities via a bidirectional cross-attention mechanism and further refines the joint representation through a residual FFN; **(d)** Prediction Output, where a MLP performs regression to estimate the on-target activity score of the input sgRNA.

To address these limitations, we propose a structurally innovative dual-branch deep neural network (Luo et al., 2024), CRISPR-FMC, which integrates MSC, Transformer encoders, BiGRU, and a bidirectional cross-attention mechanism. The architecture is designed to enhance model expressiveness along three key dimensions: diversity of input representations, coordination of feature extraction, and depth of semantic fusion (Qin and Song, 2022). As illustrated in Figure 2, CRISPR-FMC consists of four primary modules: **(a)** Pre-training (Figure 2a, b) Multimodal Processing (Figure 2b, c) Cross-modal Interaction (Figure 2c,d) Prediction Output (Figure 2d).

To effectively capture both the structural and semantic characteristics of sgRNA sequences, CRISPR-FMC adopts a dual-branch input framework (Figure 2A). This architecture enables the model to simultaneously learn from low-level compositional features and high-level contextual representations. The first branch applies conventional one-hot encoding, converting each sgRNA into a 23×4 binary matrix that preserves nucleotide-specific structural information. The second branch utilizes RNA-FM, a pre-trained RNA language model, to generate a 640-dimensional continuous embedding for each sgRNA, capturing rich semantic context. As a foundation model trained on large-scale RNA sequence corpora, RNA-FM has demonstrated strong performance in downstream tasks such as RNA secondary structure prediction and functional element annotation (Yu et al., 2024). However, its application to CRISPR-Cas9 activity modeling remains largely unexplored. The combination of discrete and continuous representations allows the model to access complementary information from distinct feature spaces, thus establishing a multi-resolution, semantically enriched foundation for downstream feature learning.

To effectively capture multi-scale sequence dependencies, CRISPR-FMC constructs separate feature extraction pipelines for the One-hot and RNA-FM branches (Figure 2B), each comprising an MSC module, a Transformer encoder, and a BiGRU module arranged sequentially. In the One-hot encoding branch, input sequences are first processed by a MSC module composed of parallel convolutional layers with kernel sizes of 1×1 , 3×3 , 5×5 , and 7×7 . This architectural design enables the model to extract local features across multiple receptive fields, thereby capturing diverse sequence motifs of varying lengths. MSC has demonstrated effectiveness across a range of domains, including traffic sign recognition (Sermanet and LeCun, 2011) and image forensics (Liu et al., 2018), and is employed here to enhance the model’s sensitivity to local structural variations intrinsic to CRISPR sequence data. The resulting multi-scale convolutional features are subsequently passed through a Transformer encoder, which employs multi-head self-attention mechanisms to capture long-range dependencies across nucleotide positions. The encoder incorporates residual connections, layer normalization, and a position-wise FFN to promote training stability and enhance representational flexibility. The Transformer output is then fed into a BiGRU module, which captures bidirectional temporal dependencies and enables context-aware sequence modeling. This Transformer–BiGRU combination has demonstrated strong performance in tasks such as text classification (Shaheen et al., 2020) and clinical time-series prediction (Song Z. et al., 2024), motivating its adoption here for joint modeling of global attention and sequential dependencies. For the RNA-FM branch, the 640-dimensional input vector is first linearly projected and reshaped, then processed by an independent Transformer–BiGRU stack. Given the semantic gap between RNA-FM embeddings and One-hot encodings, separate parameter sets are employed in the RNA-FM pathway to preserve semantic fidelity and mitigate representational interference. By maintaining distinct feature extraction paths for each modality, the model preserves their intrinsic heterogeneity and prevents premature fusion, which could otherwise lead to information entanglement or redundancy. The outputs from both branches are high-level feature tensors of shape [B,23,256] , which are forwarded to the fusion module.

To enable deep semantic alignment and effective integration of heterogeneous feature representations, CRISPR-FMC incorporates a bidirectional cross-attention mechanism within its cross-modal interaction module (Figure 2C). Feature embeddings from both branches are linearly projected into a shared space of dimension [B,23,64] and alternately serve as the *Query* and *Key/Value* components within the cross-attention framework. This mechanism allows One-hot features to absorb contextual signals from RNA-FM embeddings, and vice versa, thereby facilitating mutual enhancement and semantic coherence across modalities.

The attention-refined features are further processed via residual connections and layer normalization to stabilize training and preserve gradient flow. Bidirectional outputs are concatenated along the feature dimension to form a unified representation of shape [B,23,128] . A residual feedforward network (FFN) is then applied to introduce nonlinear transformations while preserving the original signal, enhancing feature discriminability. The fused representation is flattened and passed to a Multi-Layer Perceptron (MLP) for regression-based prediction, as shown in Figure 2d. The MLP comprises three hidden layers with 80, 40, and 20 neurons, each followed by ReLU activation and dropout layers with rates of 0.4, 0.3, and 0.2 to mitigate overfitting Taud and Mas (2017). The output layer contains a single neuron that produces the predicted on-target cleavage efficiency. Serving as the decision head of CRISPR-FMC, the MLP maps the fused features to the final prediction.

### 2.4 Experimental settings

Model training and evaluation were conducted on a local high-performance computing platform with an NVIDIA GeForce RTX 2080Ti GPU (22 GB VRAM), an Intel Core i5-12400F CPU, and 32 GB RAM, running Windows 11. The model was implemented in PyTorch 1.8.1 with Python 3.9. All dependencies, including numpy, pandas, scikit-learn, matplotlib, scipy, and fair-esm, were managed via Conda and PyPI to ensure experimental reproducibility.

Based on the datasets described in Section 2.1 and summarized in Table 1, model training and evaluation were performed independently on each of the nine publicly available CRISPR-Cas9 datasets. These datasets encompass a wide range of cell types and sgRNA activity measurements across varying experimental scales. A dual-branch architecture was employed to capture multi-level sequence representations, with the corresponding encoding strategies detailed in Section 2.2.

All target values were scaled to the [0,1] range through Min-Max normalization. A five-fold cross-validation scheme was employed with a fixed random seed (2024) to ensure reproducibility. Training was conducted with a batch size of 4,096 for up to 200 epochs. The model was optimized with the Adamax algorithm, initialized with a learning rate of $3\times 10^{-4}$ . Early stopping was applied to prevent overfitting, and training was terminated if the validation loss failed to improve for 10 consecutive epochs. The model checkpoint corresponding to the lowest validation loss was retained for final evaluation.

The regression objective was defined based on the Log-Cosh loss (Equation 1), which combines the smooth optimization characteristics of the mean squared error (MSE) Marmolin (1986) with the robustness of the mean absolute error (MAE) Chai and Draxler (2014) against outliers. The Log-Cosh loss is defined as:

$$L_{\text{LogCosh}}=\frac{1}{N}\overset{N}{\underset{i=1}{\sum }}\log\left(\cosh\left({\hat{y}}_{i}-y_{i}\right)\right),$$ (1)

where ${\hat{y}}_{i}$ denotes the predicted value, $y_{i}$ is the ground truth, and N represents the number of training samples. For comparison, the conventional MSE (Equation 2) and MAE (Equation 2) losses are formulated as:

$$L_{\text{MSE}}=\frac{1}{N}\overset{N}{\underset{i=1}{\sum }}{\left({\hat{y}}_{i}-y_{i}\right)}^{2},\;L_{\text{MAE}}=\frac{1}{N}\overset{N}{\underset{i=1}{\sum }}\left|{\hat{y}}_{i}-y_{i}\right|.$$ (2)

Compared to MSE and MAE, the Log-Cosh (Xu et al., 2020) loss behaves similarly to MSE for small residuals, providing smooth gradients, and transitions to MAE-like behavior for large residuals, thereby enhancing robustness to outliers. This property makes it well suited for biological prediction tasks that involve experimental noise or measurement variability.

Throughout training, the loss at each epoch was recorded to monitor convergence and assess training dynamics. For each cross-validation fold, the model with the lowest validation loss was selected and retained for subsequent inference.

### 2.5 Model effect evaluation

To comprehensively assess the predictive performance of CRISPR-FMC for sgRNA on-target activity, two regression-based evaluation metrics were employed: Spearman’s rank correlation coefficient (SCC) (Equation 3) (Zar, 2005) and Pearson correlation coefficient (PCC) (Equation 4) (Benesty et al., 2008). SCC quantifies the strength of a monotonic relationship between predicted and actual values, reflecting the model’s ability to preserve the relative ranking of sgRNA activity. In contrast, PCC measures the linear correlation between predicted scores and ground truth, indicating the model’s capacity to capture numerical agreement in regression.

Let $\hat{Y}=\left\{{\hat{y}}_{1},{\hat{y}}_{2},\ldots ,{\hat{y}}_{N}\right\}$ and $Y=\left\{y_{1},y_{2},\ldots ,y_{N}\right\}$ denote the predicted and true values, respectively. The evaluation metrics are defined as follows:

$$\text{SCC}=1-\frac{6\sum_{i=1}^{N}{\left(r\left({\hat{y}}_{i}\right)-r\left(y_{i}\right)\right)}^{2}}{N\left(N^{2}-1\right)}$$ (3)

where $r\left({\hat{y}}_{i}\right)$ and $r\left(y_{i}\right)$ denote the ranks of ${\hat{y}}_{i}$ and $y_{i}$ in their respective sets, and N is the total number of samples.

$$\text{PCC}=\frac{\sum_{i=1}^{N}\left({\hat{y}}_{i}-\bar{\hat{y}}\right)\left(y_{i}-\bar{y}\right)}{\sqrt{\sum_{i=1}^{N}{\left({\hat{y}}_{i}-\bar{\hat{y}}\right)}^{2}}\sqrt{\sum_{i=1}^{N}{\left(y_{i}-\bar{y}\right)}^{2}}}$$ (4)

where $\bar{\hat{y}}$ and $\bar{y}$ represent the means of the predicted and true values, respectively.

All evaluations were conducted on the test folds of five-fold cross-validation. Metric computation was performed with the scipy.stats module in Python to ensure standardized and reproducible evaluation.

## 3 Results and analysis

### 3.1 Model comparison

To rigorously evaluate the effectiveness of the proposed CRISPR-FMC model in predicting CRISPR-Cas9 sgRNA activity, we conducted comparative experiments against several state-of-the-art baseline methods across nine publicly available datasets (see Table 1 for details). The competing methods include CrnnCrispr (Zhu et al., 2024), CRISPR-ONT (Zhang et al., 2021), TransCrispr (Wan and Jiang, 2022), and C-RNNCrispr (Zhang et al., 2020), each representing a distinct network design paradigm and feature modeling strategy. To ensure a fair and reproducible comparison, all baseline results presented in this study were independently reproduced by our team. We utilized publicly available source code for each model and trained and evaluated them under the same five-fold cross-validation protocol used for CRISPR-FMC. Specifically, CrnnCrispr integrates CNN and BiGRU modules to capture local sequence motifs and contextual dependencies. CRISPR-ONT incorporates an attention mechanism to enhance the identification of key functional sites. TransCrispr employs a hybrid CNN–Transformer architecture to strengthen the modeling of long-range dependencies. C-RNNCrispr, on the other hand, adopts a recursive convolutional structure to facilitate hierarchical feature extraction across multiple semantic levels.

**FIGURE 3:**
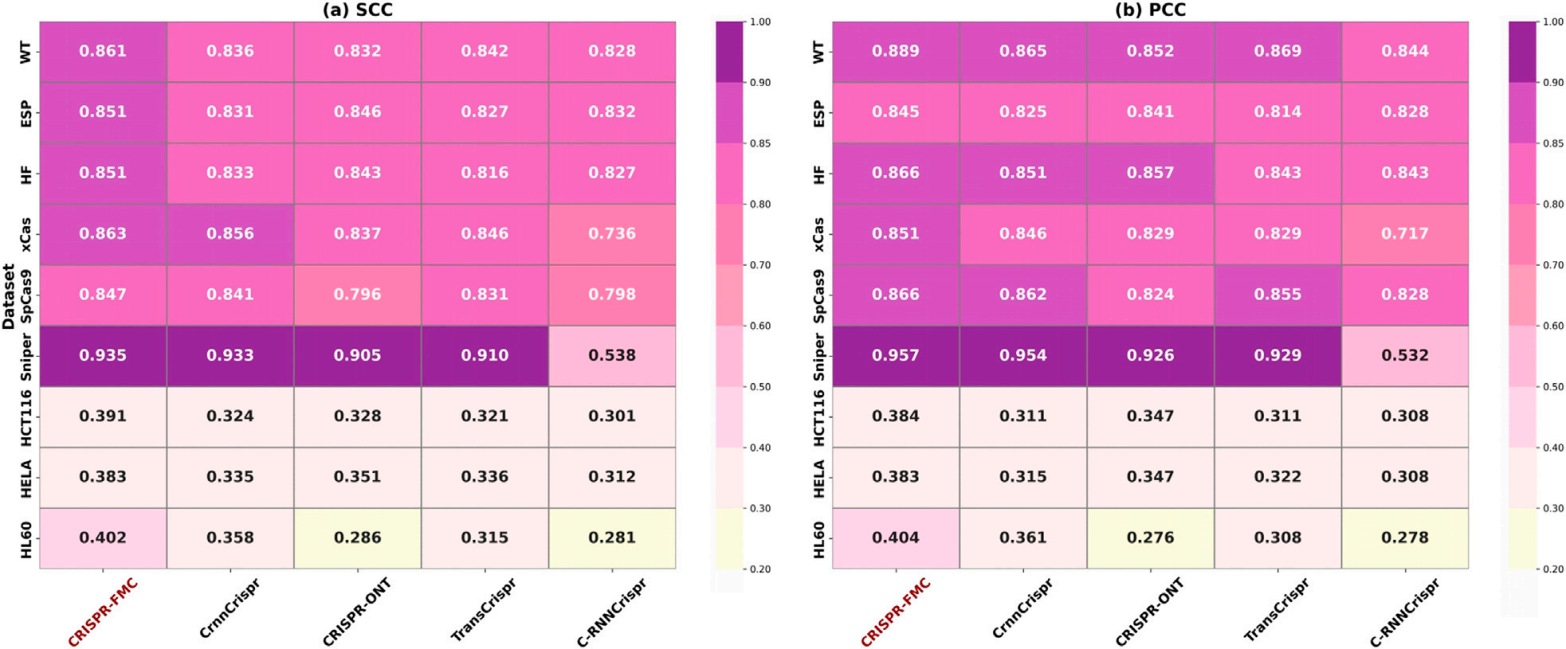
The color corresponding to the highest numerical values in both panels **(a)** SCC and **(b)** PCC has been adjusted to a darker shade to accurately reflect the color scale. Darker colors indicate stronger predictive performance. CRISPR-FMC consistently achieves superior results across most datasets in both metrics.

The experimental results are summarized in Figure 3. CRISPR-FMC consistently achieved the highest performance across all nine datasets. On large-scale datasets such as WT, ESP, and HF, the model attained SCC values of 0.861, 0.851, and 0.851, along with PCC scores of 0.889, 0.845, and 0.866, respectively—showcasing robust predictive capability in data-rich settings. The margins over the best-performing baseline (e.g., CrnnCrispr or TransCrispr) were moderate, typically ranging from 1.5% to 2.5%.

Notably, on the Sniper dataset, CRISPR-FMC achieved the highest performance with SCC = 0.935 and PCC = 0.957, outperforming the second-best model (CrnnCrispr) which scored 0.933 and 0.954, respectively. This result demonstrates the model’s effectiveness on variant-specific Cas9 datasets.

The model also performed well on small-scale datasets such as HCT116, HELA, and HL60. On the HL60 dataset, CRISPR-FMC reached SCC = 0.402 and PCC = 0.404, surpassing CrnnCrispr, which achieved SCC = 0.358 and PCC = 0.361. These results confirm that CRISPR-FMC maintains strong generalization ability even under limited data availability.

The overall performance advantage can be attributed to the model’s dual-branch architecture and multimodal feature fusion, combining One-hot encoded features and RNA-FM embeddings. The design enables the capture of both low-level sequence motifs and high-level contextual dependencies through MSC, BiGRU, and cross-attention modules, thereby enhancing prediction robustness across different datasets.

### 3.2 Ablation experiments

To systematically assess the contribution of each architectural component within the CRISPR-FMC model to sgRNA activity prediction, we performed a series of comprehensive ablation experiments across nine publicly available CRISPR-Cas9 datasets. In each experiment, a single submodule was removed or replaced, while the remaining model architecture and training protocol were kept unchanged. This controlled design ensures that any observed performance variation can be directly attributed to the specific structural modification. The key components examined are summarized in Table 2, and the selected datasets (Table 1) encompass a wide range of sequence complexities and sample sizes.

**TABLE 2:** Summary of CRISPR-FMC Variants in the Ablation Study. The average SCC and PCC values are computed across all nine CRISPR-Cas9 datasets to evaluate the impact of each architectural component.

Variant	Description
CRISPR-FMC-Onehot	Only the One-hot encoding branch is retained
CRISPR-FMC-RNAFM	Only the RNA-FM embedding branch is retained
CRISPR-FMC-w/o MSC	Replaces the MSC module with a fixed-size 5×5 convolution
CRISPR-FMC-w/o	Transformer Removes the Transformer encoder
CRISPR-FMC-w/o	BiGRU Removes the BiGRU module
CRISPR-FMC-w/o	CrossAttn Removes the bidirectional cross-attention module
CRISPR-FMC-w/o	FFN Removes the FFN

**FIGURE 4:**
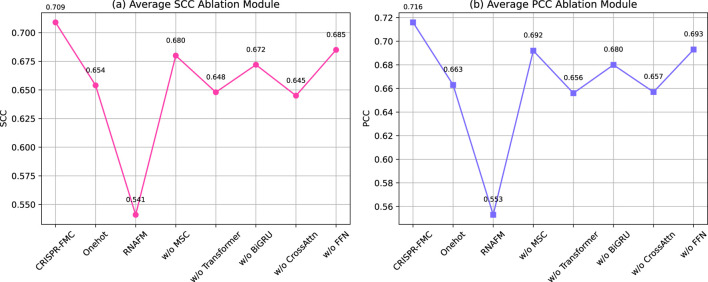
Ablation study evaluating the contribution of individual structural components to the predictive performance of the CRISPR-FMC model. The left panel displays the **(a)** Average SCC Ablation Module, while the right panel shows the **(b)** Average PCC Ablation Module. The results highlight the importance of multi-level sequence encoding and hierarchical feature extraction for accurate prediction.

As shown in Figure 4, the complete CRISPR-FMC model achieves the highest average SCC and PCC values of 0.709 and 0.716, respectively. Excluding either input encoding branch leads to notable performance degradation. Retaining only one-hot encoding still yields reduced performance (SCC: 0.654, PCC: 0.663), while relying solely on RNA-FM embeddings results in the most significant drop (SCC: 0.541, PCC: 0.553). These findings demonstrate that the two encoding schemes capture complementary aspects of sgRNA sequence information and jointly contribute to a robust feature representation.

In the sequence modeling pipeline, ablation of the MSC module (replaced with a fixed-size 5×5 convolution) reduces performance to 0.680 in SCC and 0.692 in PCC. This confirms the importance of capturing multi-scale motif patterns, particularly for datasets like HF, ESP, and WT. Similarly, removing the Transformer block results in SCC and PCC values of 0.648 and 0.656, respectively, underscoring its role in modeling long-range dependencies. The BiGRU module is also essential for contextual modeling, with its removal lowering performance to 0.672 in SCC and 0.680 in PCC. These trends align with observations from the CRISPR-MFH study by Zheng et al. (2025) and the TransCrispr model by Wan and Jiang (2022).

The cross-modal interaction mechanism is further tested via an additional variant, oCrossAttn, which retains only the bidirectional cross-attention module while removing other fusion components. This version yields lower average scores (SCC: 0.645, PCC: 0.657), indicating that although cross-attention facilitates inter-modality alignment, it cannot fully replace the complete multimodal fusion framework. Lastly, eliminating the FFN module results in SCC and PCC scores of 0.685 and 0.693, respectively, suggesting its role in refining the fused representation through high-order nonlinear transformation.

Taken together, these ablation results validate the functional necessity and complementary contributions of each structural component in CRISPR-FMC. The observed performance declines confirm the value of multi-level encoding, hierarchical feature extraction, and semantic fusion.

To further validate these findings, we conducted a detailed per-dataset ablation analysis and found that the contribution of each encoding branch varies with dataset size. Specifically, the one-hot branch dominates performance on large datasets (e.g., WT, ESP, HF), where abundant data allows the model to learn directly from sequence. In contrast, the RNA-FM branch is more effective on small datasets (e.g., HCT116, HELA, HL60), acting as a regularizer and injecting prior biological knowledge. Detailed SCC and PCC results are visualized in Supplementary Figures 6, 7, and the encoding contribution trend is highlighted in Supplementary Figure 6.

### 3.3 Generalization capability of the model

To evaluate the transferability and generalization capability of the CRISPR-FMC model across different data distributions, we conducted a cross-dataset migration experiment. Specifically, the model was independently trained on two large-scale datasets, WT and ESP, and subsequently evaluated on a small-scale dataset (HL60). The performance was assessed based on SCC and PCC. This setup simulates real-world application scenarios in which the model must generalize from well-annotated source data to novel cellular contexts characterized by limited annotations or high noise levels (Wang et al., 2020).

As shown in Table 3, CRISPR-FMC achieves superior and stable cross-dataset generalization under both training settings. When trained on the WT dataset, the model attains an SCC of 0.468 and a PCC of 0.459 on HL60, outperforming competing models such as CrnnCrispr (SCC: 0.456, PCC: 0.438) and TransCrispr (SCC: 0.448, PCC: 0.432). When trained on the ESP dataset, CRISPR-FMC still maintains top performance with an SCC of 0.448 and a PCC of 0.432, again surpassing baselines such as CRISPR-ONT (SCC: 0.432, PCC: 0.421) and C-RNNCrispr (SCC: 0.434, PCC: 0.429).

**TABLE 3:** Cross-dataset migration performance of different models trained on the WT or ESP datasets and tested on HL60. **(a)** SCC: Cross-Dataset Generalization; **(b)** PCC: Cross-Dataset Generalization.

Model	WT → HL60	ESP → HL60
(a) SCC: Cross-Dataset Generalization		
CRISPR-FMC	0.468	0.448
CrnnCrispr	0.456	0.428
CRISPR-ONT	0.454	0.432
TransCrispr	0.448	0.427
C-RNNCrispr	0.440	0.434

Model	WT → HL60	ESP → HL60
(b) PCC: Cross-Dataset Generalization		
CRISPR-FMC	0.459	0.432
CrnnCrispr	0.438	0.429
CRISPR-ONT	0.434	0.421
TransCrispr	0.432	0.424
C-RNNCrispr	0.425	0.429

Overall, CRISPR-FMC consistently achieves the highest SCC and PCC values on HL60 across both training sources, highlighting its robustness in cross-cell line migration tasks (Wang C. et al., 2014). Notably, HL60 represents a challenging test case due to its small sample size and high noise levels, yet CRISPR-FMC maintains strong predictive accuracy and correlation.

This strong generalization performance can be attributed to the architectural design of CRISPR-FMC. The dual-branch encoding scheme—combining one-hot and RNA-FM representations—enables the model to simultaneously capture local structural patterns and global semantic context. The integration of multi-scale convolution (MSC), BiGRU modules, and a bidirectional cross-attention mechanism further enhances feature expressiveness and semantic alignment, collectively improving the model’s adaptability to distributional shifts and noise.

### 3.4 Model interpretability

To gain a deeper understanding of the decision-making process of the CRISPR-FMC model, we conducted a base substitution experiment to analyze the positional sensitivity of the model’s predicted cleavage efficiency. In this experiment, the original nucleotide at each position was sequentially replaced with each of the other three bases (A, T, C, G), and the resulting changes in the model’s predictions were recorded. The impact of each substitution was quantified by calculating the Z-score (Ma et al., 2016), which reflects the magnitude of change in the predicted output.

**FIGURE 5:**
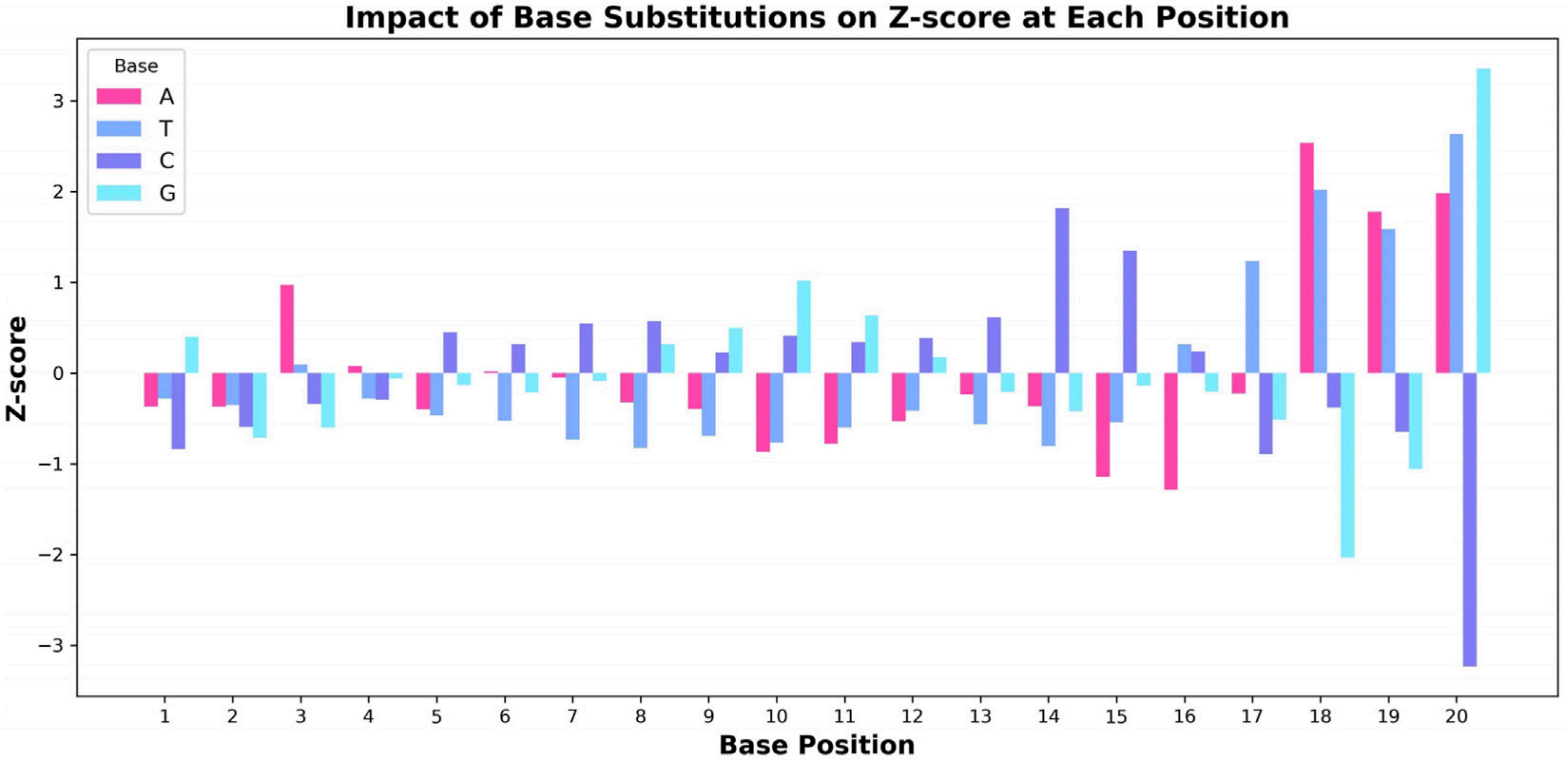
Effect of base substitution on the Z-score at each nucleotide position. The changes in Z-score resulting from substituting each base (A, T, C, G) in the sgRNA sequence are visualized, highlighting the pronounced impact of positions associated with the PAM region on predicted editing efficiency.

Figure 5 illustrates the effect of different base substitutions on the Z-score. The model exhibits the highest sensitivity in the PAM-proximal region (positions 16–20), where substitutions consistently lead to large fluctuations in the Z-score. For instance, positions 18 and 20 show particularly strong effects. Other positions, such as position three and 14, also demonstrate notable sensitivity, indicating their contribution to the model’s predictions. This observation aligns with established biological mechanisms, especially regarding the binding affinity of Cas9 to the target DNA. Previous studies, including those by Doench et al. (2016) and Duan et al. (2014), have reported that mutations in the PAM-proximal region can substantially influence Cas9 cleavage activity, reflecting the sequence-specific binding nature of the Cas9 protein.

Through this analysis, we have gained insights into how the CRISPR-FMC model prioritizes specific nucleotide positions when predicting cleavage efficiency. This understanding provides valuable guidance for optimizing sgRNA sequence design and offers a targeted strategy for enhancing CRISPR editing performance.

## 4 Contribution and discussion

This study presents CRISPR-FMC, a dual-branch deep neural network designed to accurately predict sgRNA cleavage efficiency in the CRISPR-Cas9 system. The model integrates semantic embeddings from the pre-trained RNA language model RNA-FM with conventional One-hot encoding, alongside a combination of MSC, BiGRU, and Transformer-based feature extraction modules. A bidirectional cross-attention mechanism and a FFN are incorporated to enable multimodal semantic alignment and global feature fusion. Extensive evaluations on nine publicly available datasets demonstrate that CRISPR-FMC consistently achieves higher performance scores than state-of-the-art models in both SCC and PCC metrics, exhibiting particular strengths in low-data settings and cross-dataset generalization.

The effectiveness of CRISPR-FMC stems from a synergistic and, as highlighted by our ablation studies, context-dependent integration of its components. The dual-branch encoding strategy demonstrates complementary strengths that are crucial for the model’s versatility. On large- and medium-scale datasets, performance is heavily driven by the One-hot encoding branch, which excels at capturing explicit nucleotide patterns when sufficient data is available. In contrast, on small datasets, the contribution of the RNA-FM branch becomes vital. The pre-trained RNA-FM embeddings provide rich contextual priors learned from millions of sequences, acting as an effective regularizer that guides the model toward generalizable features and prevents overfitting in data-scarce scenarios. Therefore, the architecture does not merely combine features, but adaptively leverages them—relying on robust pattern extraction in data-rich environments while harnessing pre-trained biological knowledge to ensure reliability when data is limited. This adaptability to varying data scales is a key strength, enhancing the model’s practical utility for a wide range of bioinformatics applications. Despite its promising performance, several limitations remain. A key limitation is our use of RNA-FM for sgRNA embeddings. We acknowledge the important biological nuance that the sgRNA functions within a Cas9-sgRNA ribonucleoprotein complex to target DNA, rather than as a free, unbound non-coding RNA. The target recognition process is therefore critically dependent on the properties of the target DNA sequence and its local chromatin context. This is consistent with our ablation study results, where the performance gains from RNA-FM were more modest on large-scale datasets, suggesting that DNA-level features are likely stronger determinants of cleavage efficiency. Furthermore, the scope of this study is confined to on-target activity, while predicting and minimizing off-target effects remains an equally critical challenge for safe and effective genome editing. In addition, the current web platform lacks support for high-throughput input and batch prediction, highlighting opportunities for improving deployment scalability.

Therefore, a significant future direction will be to shift the modeling focus toward the biological context of the DNA target site. Future work will focus on integrating DNA-specific language models (e.g., Nucleotide Transformer, HyenaDNA) to generate embeddings from the target DNA sequence, including flanking genomic regions. This approach would allow the model to capture features more central to the biological mechanism, such as DNA accessibility and sequence complementarity, potentially leading to substantial gains in predictive power. Beyond this, the dual-branch architecture of CRISPR-FMC could be adapted to address off-target predictions. By training the model on datasets of guide-mismatch sequence pairs, the framework could learn to predict cleavage efficiency at unintended sites, representing another promising extension of this work. We will also continue to explore incorporating other multi-omics data and uncertainty quantification techniques to improve prediction robustness. These extensions aim to provide more comprehensive and reliable decision support for sgRNA design across diverse biological contexts.

## Data Availability

The datasets and source code generated for this study are publicly available at https://github.com/xx0220/CRISPR-FMC. A lightweight web interface is also included to allow users to test CRISPR-Cas9 activity predictions by inputting sequences and selecting target datasets. Further inquiries can be directed to the corresponding authors.
