# Supplementary material for: CRISPR-FMC: a dual-branch hybrid network for predicting CRISPR-Cas9 on-target activity
Source: Front Genome Ed. 2025 Aug 29;7:1643888. doi: 10.3389/fgeed.2025.1643888 (PMC12426269; doi:10.3389/fgeed.2025.1643888)
Supplement: Supplementary file 1 [file Presentation1.pdf]

Appendix

Fig.1 and Fig.2 present detailed comparisons of the ablation results across nine CRISPR-Cas9 datasets. These results provide further insight into the contribution of each architectural module in CRISPR-FMC.

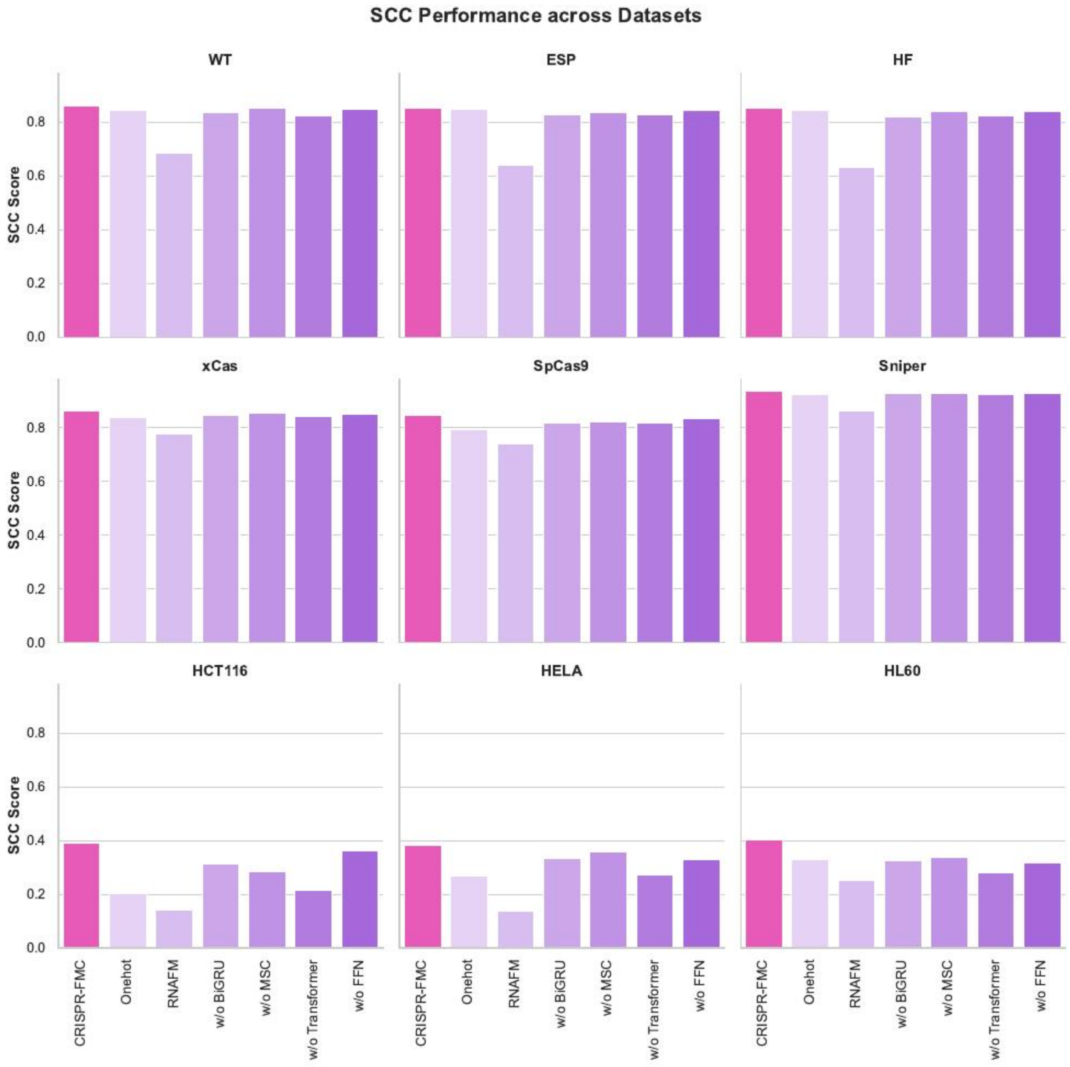

Fig 1:Ablation study results across nine CRISPR-Cas9 datasets based on SCC. Each subplot corresponds to a specific dataset, comparing the full CRISPR-FMC model against its ablated variants, including models with individual components (e.g., BiGRU, MSC, Transformer, FFN) removed or replaced. The results demonstrate that removing any single module leads to a consistent drop in performance, with particularly pronounced declines on small-scale datasets such as HELA and HL60.

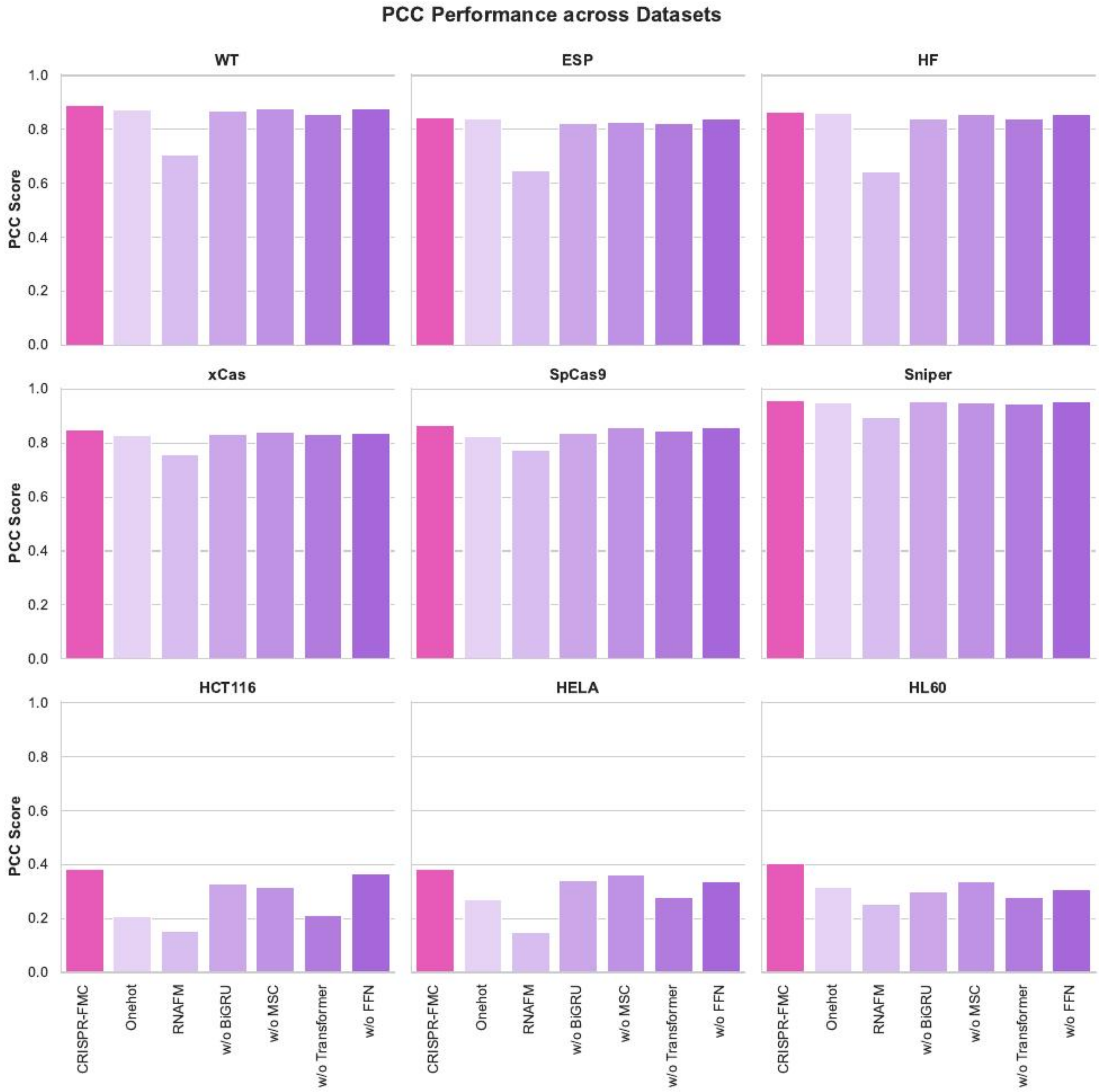

Fig 2:Ablation study results across nine CRISPR-Cas9 datasets based on PCC.
